# Combining Electrospinning and Vapor-Phase Polymerization for the Production of Polyacrylonitrile/ Polypyrrole Core-Shell Nanofibers and Glucose Biosensor Application

**DOI:** 10.3389/fchem.2020.00678

**Published:** 2020-08-04

**Authors:** Eleni Sapountzi, Jean-François Chateaux, Florence Lagarde

**Affiliations:** ^1^Univ Lyon, CNRS, Univ Claude Bernard Lyon 1, Institute of Analytical Sciences, Villeurbanne, France; ^2^Univ Lyon, Univ Claude Bernard Lyon 1, CNRS, Institut des Nanotechnologies de Lyon, Villeurbanne, France

**Keywords:** core-shell nanofibers, electrospinning, vapor phase polymerization, polyacrylonitrile, polypyrrole, glucose oxidase, impedimetric biosensor

## Abstract

In this work, polyacrylonitrile (PAN) nanofiber mats coated with conductive polypyrrole layers were produced at the surface of gold electrodes by a two-step approach combining electrospinning and vapor phase polymerization. In the first step, smooth and uniform PAN fibers exhibiting an average diameter of 650 ± 10 nm were generated through electrospinning of 12 wt% PAN solutions. The electrospun PAN fibers were impregnated with iron(III)tosylate (FeTos), annealed at 70°C and used as a robust and stable template for the growth of a thin layer of conductive polymer by co-polymerizing pyrrole (Py) and pyrrole-3-carboyxylic acid (Py3COOH) vapors under nitrogen atmosphere. The carboxyl groups introduced in polypyrrole coatings enabled further covalent binding of a model enzyme, glucose oxidase. The effect of different parameters (concentration of FeTos into the immersion solution, time of polymerization, Py/Py3COOH molar ratio) on the PAN/PPy/PPy3COOH/GOx impedimetric biosensor response was investigated. In the best conditions tested (immersion of the PAN fibers into 20 wt% FeTos solution, polymerization time: 30 min, 1:2 Py/Py3COOH ratio), the biosensor response was linear in a wide range of glucose concentration (20 nM−2μM) and selective toward ascorbic and uric acids. A very low limit of detection (2 nM) compared to those already reported in the literature was achieved. This value enables the determination of glucose in human serum after a large dilution of the sample (normal concentrations: 3.6 mM−6.1 mM range).

## Introduction

Among the various intrinsically conducting1 polymers (ICPs) prepared to date, polypyrrole (PPy) is one of the most widely used in biosensing applications, owing to its biocompatibility, high hydrophilic character, high stability at ambient conditions and ease of synthesis (Inzelt, 2018). The polymer can be combined with nanomaterials to provide enhanced properties or functions (Jain et al., 2017; Naveen et al., 2017; Naseri et al., 2018; Wang et al., 2018). In conventional electrochemical biosensors, PPy is grown as thin films onto an electrode surface via oxidative electropolymerization. Biosensing molecules, e.g., enzymes, are immobilized by direct adsorption on the polymer, cross-linking or chemical grafting on functional PPy, e.g., poly(3-carboxyle pyrrole). In a second strategy, enzymes are entrapped within the film in a simple one-step procedure performed under mild conditions thereby preserving enzyme activity. This approach also helps preventing enzymes from leaching out of the biosensing membrane. However, the enzymes are embedded in the polymeric matrix, which limits their accessibility to the substrate. Grafting the biomolecules at the electrode surface improves the accessibility.

In recent years, there have been a growing interest in developing PPy nanostructures (e.g., nanospheres, nanowires, nanocapsules, nanotubes, nanofibers,…) for sensors and biosensors applications. The structures, arranged in a more or less organized way at the electrode surface, offer smaller dimensions, larger specific surface areas and higher reactivity compared to thin films. Biomolecules loading, interaction processes and mass transfer kinetics are significantly improved, conferring enhanced sensitivity and response time to the biosensors (Xia et al., 2010; Yoon, 2013; Nguyen and Yoon, 2016; Park et al., 2016). The synthesis of PPy nanostructures may be accomplished through hard-template, soft-template or physical methods (Xia et al., 2010). Among the physical approaches, electrospinning enables for the rapid and easy generation of long, continuous nanofibers (NFs) directly on electrode surfaces without the need of post-treatment processes (Sapountzi et al., 2017). In a general way, ICPs are too stiff to be electrospun directly. Blending with a carrier polymer is usually required to improve the spinability of ICPs, to the detriment of the conductive properties of resulting NFs (Sapountzi et al., 2017; Migliorini et al., 2018). Thus, an alternative can be used to produce conductive NF mats, in which electrospun fibers of a non-conducting polymer, e.g., polyacrylonitrile (PAN) (Laforgue and Robitaille, 2010a; Ekabutr et al., 2013; Liu et al., 2016; Cetin and Camurluz, 2017), poly(ε-caprolactone) (PCL) (Guler et al., 2015), polyamide 6 (PA6) (Granato et al., 2009; Wang et al., 2013), polystyrene (PS) (Nair et al., 2008; Wang et al., 2012), or polyethyleneoxide (PEO) (Nair et al., 2005), serve as a template for ICP growth. Coating of the non-conductive backbone NFs with CP such as PPy can be achieved by electropolymerization (Wang et al., 2013) or by chemical polymerization, starting from Py monomer in solution (Guler et al., 2015; Liu et al., 2016) or in vapor form (Nair et al., 2005, 2008; Granato et al., 2009; Laforgue and Robitaille, 2010a; Wang et al., 2012; Ekabutr et al., 2013; Cetin and Camurluz, 2017).

Herein, we propose an original and efficient electroactive platform for electrochemical biosensing, based on conducting core-shell NFs produced by the combination of electrospinning and vapor phase polymerization (VPP) process. To do so, PAN, a well-studied polymer with good stability and mechanical properties, was electrospun to fabricate PAN NFs (Nataraj et al., 2012). These NFs were used as backbone non-conductive structure (core fibers), whilst facilitating the growth of PPy based coatings onto their surface. Py polymerization was performed chemically after immersion of PAN fibers into an oxidative iron(III)tosylate (FeTos) solution. Glucose oxidase (GOx) and electrochemical impedance spectroscopy were used respectively as model sensing enzyme and transduction mode to evaluate biosensing capacities of the plaform. In order to secure GOx immobilization, the enzyme was covalently bound to carboxyle groups introduced in the polypyrrole coating by copolymerizing pyrrole-3-carboyxylic acid (Py3COOH) with Py. Carboxylated PPy are excellent candidates for electrochemical biosensors because they offer suitable interface for covalent grafting of biomolecules, which results in good stability and high immobilization density. They have been studied, mostly as thin films and only by a few research groups, for the development of immunosensors (Puri et al., 2014; Iordanescu et al., 2018) or aptasensors (Jun et al., 2014; Sheikhzadeh et al., 2016). Kausaite-Minkstimiene et al. recently proposed amperometric glucose biosensors based on GOx immobilized on PPyCOOH particles and PPyCOOH particles/gold nanoparticles composites (Kausaite-Minkstimiene et al., 2018, 2020). Only two enzyme biosensors have been reported for glucose detection based on NFs and pure PPy coating, GOx being adsorbed or cross-linked at the conducting NFs surface (Ekabutr et al., 2013; Cetin and Camurluz, 2017). To the best of our knowledge, this is the first report of an enzyme biosensor where sensing biomolecules are conjugated with carboxylated Py-coated NFs prepared by combining electrospinning and VPP of Py/Py3COOH mixtures.

## Materials and Methods

### Reagents

Polyacrylonitrile (PAN, average M_w_:150000), Fe(III) p-toluenesulfonate hexahydrate, (FeTos, technical grade), 1-butanol (ButOH, 99.5%), acetonitrile (ACN, 99%), *N,N*-dimethylformamide, (DMF, 99.8%), glutaraldehyde (GA, 25 wt% aqueous solution), N-hydroxysuccinimide (NHS, 98%), *N*-(3-dimethylaminopropyl)-*N*′-ethylcarbodiimide hydrochloride (EDC, crystalline), pyrrole-3-carboxylic acid (Py3COOH, >96%), pyrrole (Py, 98%), D-(+)-glucose (>99.5%) and lyophilized glucose oxidase (GOx, type X-S, from *Aspergillus niger*, 100-250 kU/g solid) were purchased from Sigma-Aldrich (Saint-Quentin-Fallavier, France). Prior to use, Py was purified by distillation over calcium hydride under reduced pressure. Phosphate buffer saline solutions (PBS, pH 7.2, 0.1M) were prepared from monopotassic and dipotassic phosphate salts, sodium and potassium chlorides from Sigma Aldrich.

### Preparation of the Working Electrodes

One cm^2^ square gold electrodes (300-nm gold/30-nm titanium on silicon substrate) were used as working electrodes. They were fabricated by the Laboratory of Analysis and Architecture of Systems (LAAS, Toulouse, member of the French RENATECH network) using standard silicon technologies. Before modification, the gold electrodes were cleaned in an ultrasonic bath for 10 min in acetone and dried under a N_2_ flow, then dipped for 2 min at room temperature into a H_2_O_2_:H_2_SO_4_ (3:7 v/v) piranha solution and rinsed with ethanol. Between and after these treatments, the gold electrodes were rinsed thoroughly with ultrapure water.

### Preparation of Electrospun Solutions

#### PAN Solutions

For the fabrication of PAN NFs, 7, 12, and 18 wt% PAN solutions were prepared by dissolving adequate amounts of PAN powder into DMF at 80°C for 3 h under magnetic stirring. The solutions were cooled down to room temperature before use.

#### PAN/FeTos Suspensions

For the fabrication of PAN/FeTos NFs, a 15 wt% PAN solution was prepared by dissolving PAN powder into DMF at 80°C for 5 h under magnetic stirring. Three different FeTos solutions (12, 20 and 40 wt% in DMF) were also prepared and added to the PAN solution individually. The resulting PAN/FeTos solutions were heated at 80°C for 3 h under magnetic stirring. Different mass ratios of the PAN and FeTos solutions (1:1, 1:2, 2:1 and 3:1, respectively) were tested for each concentration of the oxidant.

### Electrospinning

NFs were fabricated using a home-made electrospinning device. The spinneret was made of a glass syringe equipped with a luer lock port to connect blunt-end needles. To control the feed rate of the polymer solution with no induced fluctuations, a pneumatic jack driven by an electro-pneumatic regulator (ITV 1030, SMC) and controlled through a LabView interface was used. The electrical field was produced by a high voltage power supply (Spellman CZE1000R) delivering up to 30 kV.

PAN and PAN/FeTos solutions were loaded immediately after preparation into a glass syringe fitted with a stainless needle (0.644 mm I.D.), the cleaned working gold electrodes were placed on the collector inside the ES chamber and fibers were spun at room temperature (23 ± 2°C). The nanofibers were directly deposited on the surface of the gold electrodes. Electrospinning parameters (e.g., applied voltage, feed rate and collection distance) ranging from 8 to 22 kV for the applied voltage, 9 to 23 cm for the tip-to-collector distance and 1–3 mL/h for the polymer flow rate, were adjusted to limit bead formation.

### VPP Process

**Figure 1:**
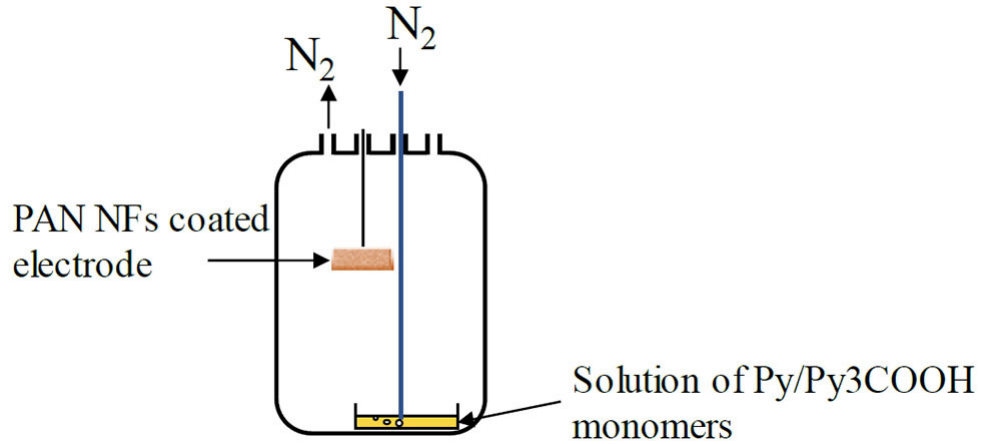
VPP coating process.

The PAN NFs coated electrodes were dipped into an oxidant FeTos solution (12, 20, or 40 wt% in ButOH). Spincoating for 20 s at 3000 rpm allowed removing the excess of FeTos. The FeTos-impregnated NFs were then annealed for 5 min in air at 70°C on a hot plate and introduced in a reactor containing the liquid monomer(s) (Py, Py3COOH, or a mixture of both). The monomer(s) vapors, generated by bubbling nitrogen into the solution, polymerized when they came in contact with the FeTos-impregnated NFs, producing a conductive polymer coating doped with tosylate anions at the electrode surface (Figure 1).

In the PPy (resp. PPy3COOH) coating procedure, a 0.05M Py (resp. Py3COOH) solution was prepared by appropriate dilution of the monomer in acetonitrile and placed into the reactor. In the PPy/PPy3COOH coating procedures, the Py solution was mixed with a 0.05 M or 0.1 M PPy3COOH solution prepared in acetonitrile and allowed to evaporate. The polymerization time for PPy and PPy/PP3COOH mixtures were set at 15 and 30 min, respectively. After polymerization, the PAN/FeTos/PPy and PAN/FeTos/PPy/PPy3COOH NFs were removed from the reactor and left in ambient atmosphere for 4–5 h to ensure complete evaporation of Py and Py/Py3COOH vapors. They were then ultrasonicated for 1 min, rinsed with H_2_O/MeOH mixture (1:1 v:v) for 10 min and dried under N_2_ at room temperature.

### Immobilization of GOx

#### Immobilization on PPy Coated PAN NFs

One hundred μL of a mixture containing 5 mg of GOx and 5 mg of BSA were prepared in phosphate buffer (20 mM, pH 7.2). From this mixture, 20 μL were deposited onto the PAN/PPy modified gold electrode surface by spin coating for 15 s at 500 rpm and then for 15 s at 1,000 rpm. Then, the electrodes were exposed to GA saturated vapors for 30 min. GA allows the cross-linking of GOx and BSA. BSA, a lysine-rich protein with no enzymatic activity, was used as cross-linking co-reagent to help forming enzyme immobilization matrices and protect the enzyme from excessive reaction with GA, which might have compromised its activity (Barbosa et al., 2014). Finally, the biomembranes formed on top of the electrodes were dried at room temperature for 1 h and kept dry overnight at 4°C.

#### Covalent Immobilization of GOx Onto PPy/PPy3 COOH Coated PAN NFs

One hundred mM stock solutions of EDC and NHS were prepared by dissolving respectively 1.9 mg EDC and 1.2 mg NHS in 100 μL of PBS. Subsequently, the two solutions were mixed together and diluted to achieve a final solution containing 10 mM of both reagents. The gold electrodes modified with PAN/PPy/PPy3COOH NFs were immediately immersed into the EDC/NHS mixture for 1 h. Afterwards, the activated surface of the PAN/PPy/PPy3COOH NFs surface was washed 3 times with distilled H_2_O to remove the excess of unreacted EDC/NHS. Finally, the modified working electrodes were immersed into the enzyme solution (10 mg GOx in 300 μL of 5 mM phosphate buffer, pH 7.2) for 2 h at room temperature, washed with distilled H_2_O and immediately used for electrochemical measurements.

### Electrochemical Measurements

Characterizations by electrochemical impedance spectroscopy (EIS) were all performed at room temperature with a Voltalab 80 PGZ 402 analyzer (Hach Lange, France) using a 5 mL glass cell placed into a Faraday cage. The electrochemical cell was equipped with three electrodes, including the NFs modified gold working electrode (active surface: 0.07 cm^2^), a Ag/AgCl reference electrode and a Pt plate counter electrode (active surface: 0.29 cm^2^). Active surfaces of the working and counter electrodes were delimited via o-ring seals used to fit the electrodes with the measurement cell.

The frequency was varied in the 100 mHz to 100 kHz range, acquiring 5 points per decade. An excitation voltage of 10 mV was superimposed on a dc potential of −300 mV vs. Ag/AgCl.

Biosensing experiments were performed by injecting small aliquots (typically 5–200 μL) of concentrated glucose solutions into the electrochemical cell containing either PAN/ PPy/GOx NFs or PAN/ PPy/PPy3COOH/GOx NFs modified electrodes and 0.1 M PBS pH 7.2 as electrolyte. The calibration curve was built in the 20 nM-2 μM glucose range by plotting Z'-Z'_0_ values obtained at 1.6 Hz vs. log[glucose], where Z' is the real part of the impedance signal and Z'_0_ refers to Z' value for [glucose] = 0. Three replicates were performed for each glucose concentration and related standard deviations were calculated.

### Characterization of NFs Morphology

NF mats were characterized by transmission electron microscopy (TEM) using a Philips CM120 instrument operating at an accelerating voltage of 120 kV and by scanning electron microscopy (SEM) with a TESCAN MIRA3 FEG-SEM microscope after sample metallization (2 nm Pt or Cr).

## Results and Discussion

### Fabrication of PAN Electrospun NFs

**Figure 2:**
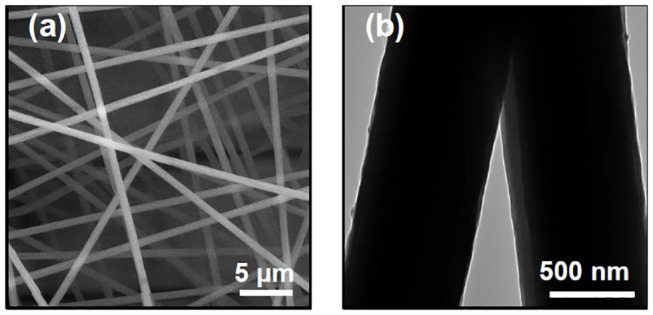
SEM **(a)** and TEM **(b)** images of electrospun PAN NFs formed by using an applied voltage of 22 kV, a polymer concentration of 12 wt%, flow rate of 1 mL/h and collection distance of 15 cm.

The influence of PAN concentration on NFs morphology was first investigated. Three concentrations, i.e., 7, 12, and 18 wt% of PAN, were tested. We observed that it was not possible to produce NFs at the lowest polymer concentration due to the insufficient viscosity of the solution for ES. Increasing PAN concentration from 7 up to 12 wt% helped solving this issue and pure fibers were generated. The 18 wt% PAN solutions appeared as hardly electrospinnable due to the very high viscosity that they exhibited. The 12 wt% concentration was therefore selected for further experiments. The influence of applied voltage (ranging from 8 to 22 kV), the tip-to-collector distance (9–23 cm) and the polymer flow rate (1–3 mL/h) on NFs morphology were further investigated. It was observed that when the applied voltage was fixed at 22 kV, the collection distance at 15 cm and the flow rate was set at 1 mL/h, the ES process was very stable and allowed the continuous production of very uniform PAN NFs, free of beads with an average diameter of 677 ± 20 nm (Figure 2). TEM images of the pure PAN NFs revealed a perfectly smooth fiber surface.

### Fabrication of Oxidant PAN NFs

Two different routes were investigated to produce oxidant electrospun PAN NFs. In the first approach, fibers were generated by electrospinning mixtures of PAN and oxidant. In the second approach, pure PAN electrospun NFs used as backbone core fibers were first produced and then immersed into the oxidant solution. FeTos and FeCl_3_ are commonly used as oxidant, but Nair et al. (2008) showed that FeTos is more adequate than FeCl_3_ for further polymerization of Py, because it results in a more rapid growth and cristallinity of PPy, and therefore to higher conductivity.

In the first set of experiments where PAN/FeTos mixtures were electrospun, three polymer/oxidant blends were prepared by mixing equal masses of a PAN solution (12 wt% in DMF) and a FeTos solution containing 12, 20, or 40 wt% of oxidant in DMF. The mixtures exhibited insufficient viscosity for electrospinning but this issue could be overcome by increasing PAN concentration from 12 to 15 wt%.

Further electrospinning tests were carried out by varying the mass ratios (i.e., 1:1, 1:2, 2:1, and 3:1) of the 15 wt% PAN solution and the three FeTos solutions (12, 20, 40 wt% FeTos in DMF). Only the polymer/oxidant mixtures with 1:1 and 2:1 ratios exhibited the required viscosity whereas the viscosity of the solution by using 3:1 ratio became so high that after the addition of the polymer a gel formed at room temperature, leading to the solidification of the mixture. On the contrary, when a ratio of 1:2 was used, the viscosity of the resulting mixture was insufficient to obtain stable conditions for electrospinning.

Electrospinning of all the 1:1 mixtures, regardless of FeTos concentration, resulted in the production of PAN/FeTos NFs exhibiting the characteristic yellow/orange color of Fe^III^ salts. However, it was hard to achieve highly stable jets and control all the parameters in order to deposit the NFs regularly onto the gold surface. Above 15 kV, sparks creation made the process impossible. This result may be attributed to the electrical conductivity of FeTos solutions (σ = 0.52 mS/cm for 40 wt% salt content, from Laforgue and Robitaille, 2010b). So, in order not to compromise the reproducibility of the glucose sensor, it was preferred to produce pure PAN NFs and then immerse them into the oxidant solution.

### Fabrication of PPy and PPy3COOH Coated PAN NFs

Using this approach, PAN electrospun NFs were produced in the optimal conditions defined above and immersed in FeTos solutions (12, 20, or 40 wt%). The excess of oxidant was removed by centrifugation using a spincoater and, after annealing at 70°C, the VPP process was initiated. First experiments were carried out by evaporating solutions of pure monomers (0.05 M of Py or PPy3COOH).

Uncoated PAN NFs exhibited a white color (Figure S1A), whereas PAN NFs immersed in FeTos solution and annealed at 70°C, exhibited the characteristic yellow/orange color of the oxidant solution (Figure S1B). PAN NFs successfully coated with PPy or PPy3COOH displayed a black color, characteristic of the presence of the polymers (Figure S1C).

#### PPy Coated PAN NFs

Whatever the concentration of FeTos solution tested, Py polymerized rapidly at ambient temperature. The characteristic black color of PPy could be detected within a few minutes only. The whole membrane looked very uniform, without any color variations and physical defects.

##### SEM characterization of PPy coated PAN NFs

**Figure 3:**
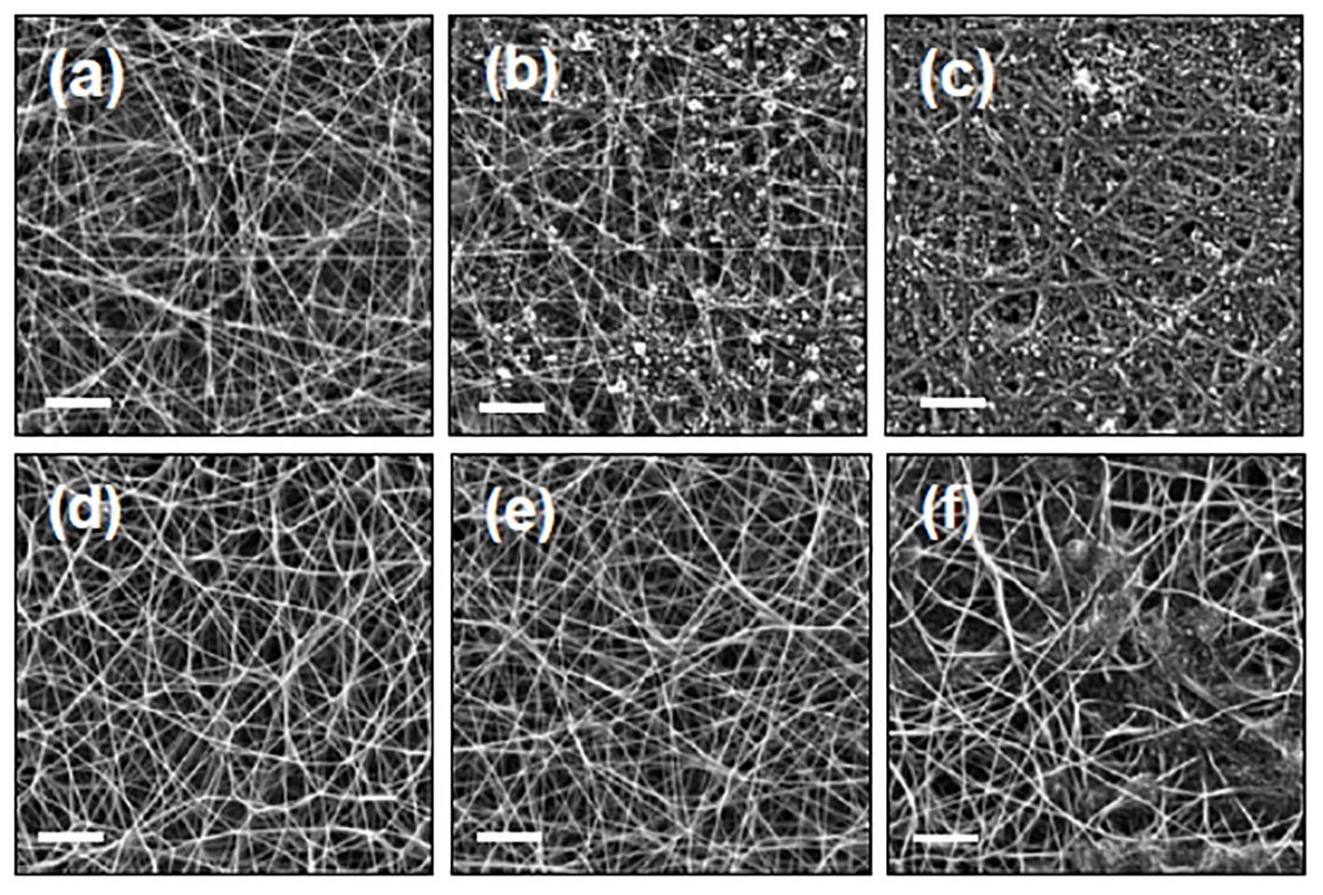
SEM images of PAN NFs covered with PPy coatings before **(a–c)** and after **(d–f)** the cleaning step. The PAN NFs mats were immersed in FeTos 12 wt% **(a,d)**, 20 wt% **(b,e)** or 40 wt% **(c,f)**, respectively, before VPP process. Scale bars represent 5 μm.

Figures 3a–c shows typical SEM images of PAN/PPy NFs produced by VPP process after immersion of the PAN NFs into 12, 20, and 40 wt% FeTos solutions, respectively. The images reveal the presence of FeTos crystals within the interfiber porosity of the mats, the amount of which increasing upon increasing the concentration of the oxidant solution. This could not be attributed only to FeTos concentration in itself, but also to the highest viscosity of the solution that prevented an efficient removal of the excess of oxidant from the mat during the spincoating process. In order to restore the porosity of the mats and then favor further contact with sample solutions, different speed rates ranging from 0 to 3,000 rpm were tested during the spincoating process. It was found that removal was maximal at 3,000 rpm but remained partial. Thus, an additional cleaning step was necessary. Different solvents were tested including distilled H_2_O, pure methanol, ethanol and a mixture of H_2_O/methanol (1:1) which was found to be the most efficient. Simply rinsing the modified electrodes was not sufficient to remove the totality of FeTos crystals, so the PAN/PPy NFs modified gold electrodes were first placed into an ultrasonic bath. The sonication time was varied from 0 to 3 min. For sonication times <1 min, the removal of unreacted FeTos was incomplete, independently of oxidant concentration, and for sonication times higher than 3 min, partial removal of the fibers themselves was observed. Thus, optimum conditions were found at ultrasonication time of 1 min followed by rinsing with H_2_O/MeOH.

SEM images presented in Figures 3d–f confirm the total elimination of crystallized FeTos salts when FeTos solutions of 12 and 20 wt% are used. The successful removal of FeTos salts after rinsing NFs with methanol has been also reported and confirmed by X-ray diffraction analysis by some authors (Laforgue and Robitaille, 2010b). At the 40 wt% concentration (Figure 3f), only partial removal of FeTos crystals was achieved, also confirming some previous results reported in the literature (Laforgue and Robitaille, 2010a). Thus, this concentration was not tested in further experiments.

**Figure 4:**
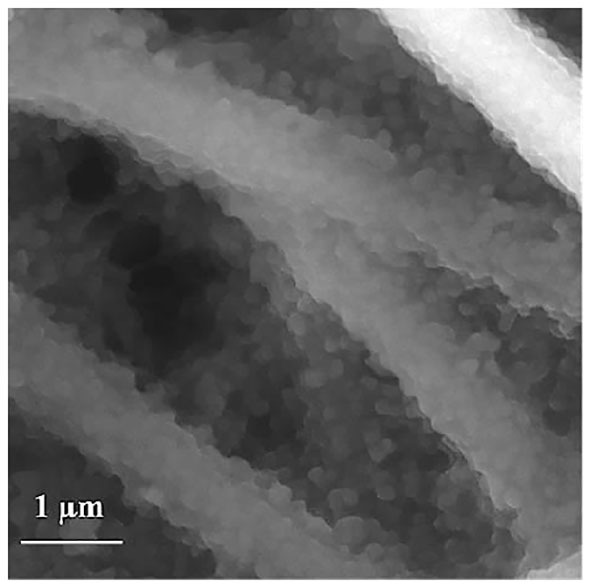
SEM images of PAN NFs coated with PPy after exposure to Py vapors for 15 min in ambient conditions. 20 wt% FeTos.

SEM images also confirmed the successful polymerization of Py on the PAN NFs surface. The formation of typical cauliflower shaped PPy nanostructures was observed and a complete coverage of the surface was achieved after 15 min of polymerization, independently of FeTos concentration (Figure 4).

##### Electrochemical characterization of PPy coated PAN NFs

EIS technique was further employed to evaluate the electrochemical properties of gold electrodes modified with PAN/PPy NFs upon increasing concentration of the oxidant solution. EIS is a label-free, sensitive and non-destructive technique particularly well suited to characterize the electrical properties of electrode/electrolyte interfaces and investigate biological events, e.g., enzyme reactions, occurring at the modified electrode surfaces (Bahadir and Sezgintürk, 2016). In EIS technique, a small sinusoidal perturbation is applied to the modified electrode polarized at a constant potential. This enables for the acquisition of signals, which are linear in time and well suited for analytical purposes (Ramanavicius et al., 2014). EIS spectra were recorded in the 100 mHz to 100 kHz frequency range by applying an AC voltage of −300 mV vs. Ag/AgCl and 10 mV amplitude. Different potentials were tested in the −500 mV vs. Ag/AgCl to 500 mV vs. Ag/AgCl range. −300 mV vs. Ag/AgCl allowed minimizing the charge transfer resistance of the modified electrodes and this negative potential is also favorable to minimize some common interferences in serum samples, e.g., ascorbic and uric acids. It was therefore considered as the most appropriate for analytical application purpose.

**Figure 5:**
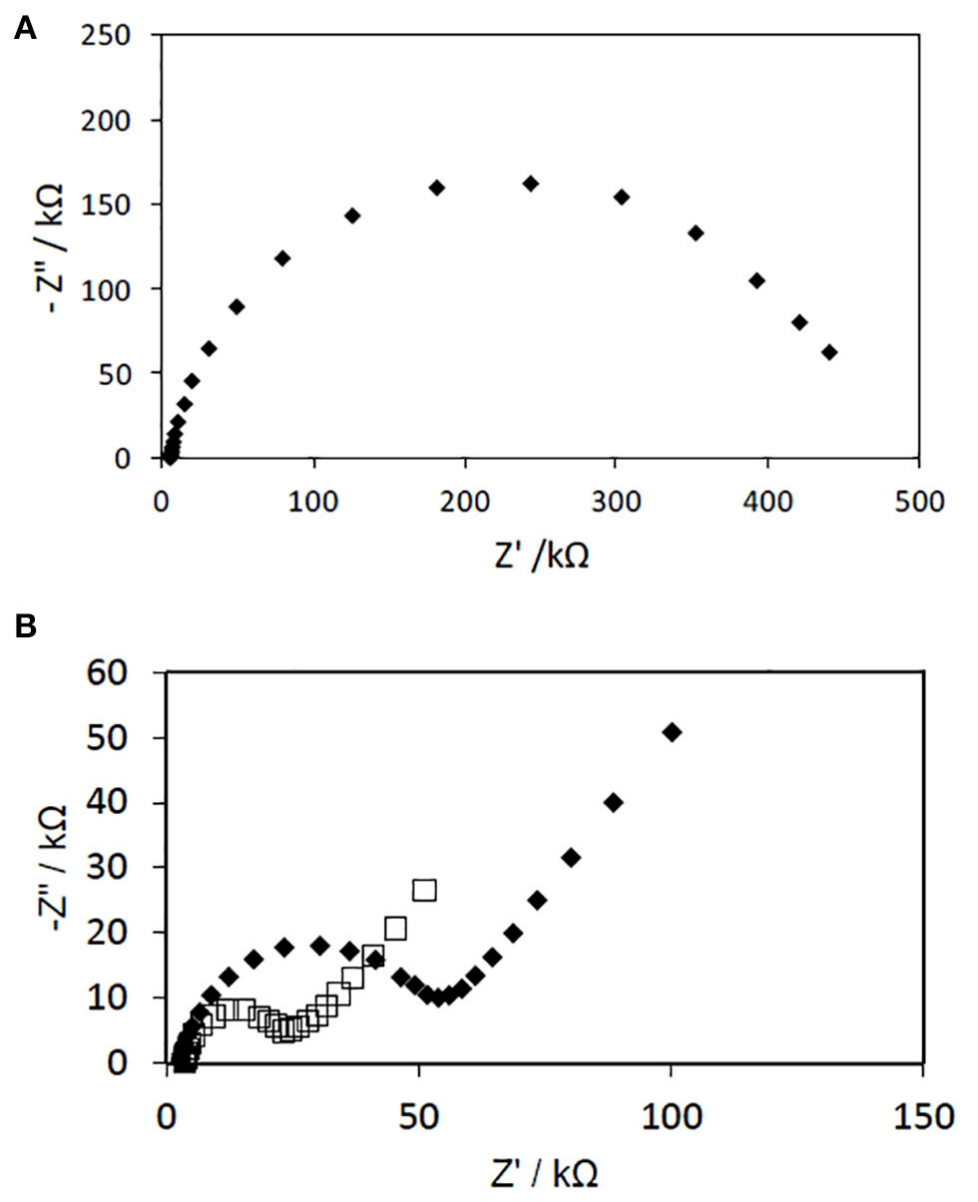
Nyquist plots of impedance spectra obtained for gold electrodes modified with PAN NFs **(A)** and PAN/PPy **(B)**. The PAN NFs were dipped into a 12 wt% (♦) or 20 wt% (□) FeTos solution, spin-coated and cleaned before reaction with 0.05 M Py vapor for 15 min. The EIS measurements were performed at −300 mV vs. Ag/AgCl in the presence of PBS solution by varying frequency in the 100 mHz to 100 kHz range.

The Nyquist plots of PAN NFs electrodes before and after coating with PPy (polymerization time: 15 min) are presented in Figure 5. Both spectra (12 and 20 wt% FeTos) exhibited a slightly distorted semi-circle. The charge transfer resistance of the NFs mat, directly related to the diameter of the semi-circle, was divided by a factor of about 10 and 20 by coating the fibers with PPy, and by increasing FeTos concentration from 12 to 20 wt%, respectively. The first result confirms that PPy acts as a good charge transferring agent. The second is consistent with the slight increase of the PPy layer thickness, as observed by SEM (data not shown). This may be also due to the presence of small quantities FeTOs salts, which were not totally removed by the washing step, this quantity increasing with initial FeTOs concentration. The Spectra recorded for the PAN/PPy NFs exhibited also a linear part in the low frequencies range, characteristic of diffusion-limited processes within the NFs mat.

We tried to model these impedance spectra as well as all the spectra presented in the following sections, using first the classical Randles model, and then some other more complex models, but fitting results were not totally satisfactory. As our main objective was to demonstrate the feasibility of our approach to develop operational biosensors using GOx as model enzyme, and not to find a suitable equivalent circuit able to describe the electrochemical system, we did not concentrate our efforts on modeling.

Experimental data at other potentials, such at 0 mV vs. Ag/AgCl, would be more informative for a better description of the system (Ramanavicius et al., 2014), but less interesting from an analytical application point-of-view.

After cross-linking GOx on the PPy coated NFs, glucose was injected at different concentrations. A large number of electrodes were tested, but in all cases Nyquist plots obtained at the different glucose concentrations were superimposed with the curve recorded before glucose injection.

After the removal of the working electrode from the electrochemical cell, the removal of the membrane containing the biomolecule of interest (GOx) was certified, whilst the PAN/PPy NFs were still well attached to the gold substrate. The first protocol tested for the production of PAN/PPy/GOx NFs modified electrodes was therefore not satisfying since it did not enable a firm attachment of the biological sensing membrane onto the fibers. This is why a second strategy of enzyme immobilization was investigated, which consisted in the covalent binding of GOx by incorporating functional carboxylic groups to the PPy coating. This was done by incorporating PP3C containing carboxylic groups in the conductive layer covering the PAN NFs.

#### PPy3COOH Coated NFs

Similar results were obtained by coating PAN NFs with PPy3COOH, except that longer polymerization times were required. The black characteristic color of the polymer coating started to appear only after 20 min, which may arise from slower evaporation and/or reaction processes.

Considering this difference in Py and Py3COOH evaporation/reaction rates, further experiments where the coating of PAN NFs with PPy/PPy3COOH composites was considered, were performed using polymerization times above 30 min.

### Fabrication of Electrospun PAN NFs Coated With PPy/PPy3COOH Composites

In a first set of experiments, VPP step was carried out by using an acetonitrile solution containing an excess of Py monomer bearing carboxyle groups (2:1 Py3COOH/Py molar ratio) to favor subsequent grafting of the enzyme. 12 and 20 wt% FeTos concentrations were tested.

In all cases, the nanofibrous mat turned gray/black after 30 min polymerization time. The whole membrane looked very uniform, without any color variations and physical defects. SEM images revealed that PPy/PPy3COOH thin coating was grown onto the surface of oxidant PAN NFs and that all the coated NFs maintained their fibrous morphology after the cleaning step.

The electrochemical properties of the PAN/PPy/PPy3COOH NFs modified gold electrodes were further characterized by EIS in the same conditions as those used for PAN/PPy NFs.

**Figure 6:**
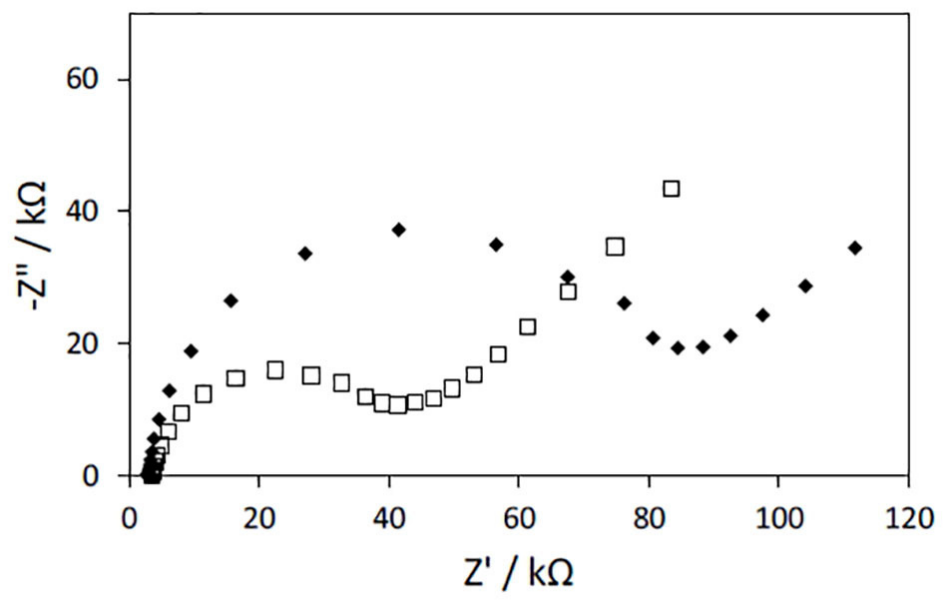
Nyquist plots of impedance spectra obtained for gold electrodes modified with PAN/PPy/PPy3COOH NFs. PAN NFs were dipped into 12wt% (♦) or 20 wt% (□) FeTos solutions and (1:2) Py/Py3COOH solutions were used for VPP (polymerization time: 30 min). The EIS measurements were performed at −300 mV vs. Ag/AgCl in the presence of PBS solution by varying frequency in the 100 mHz to 100 kHz range.

Impedance measurements of PAN/PPy/PPy3COOH NFs modified electrodes revealed a 2-fold increase in the charge transfer resistance when compared to the PAN/PPy NFs modified electrodes fabricated under the same conditions (Figure 6). This result is consistent with previous works and may be attributed to the alteration of π-π conjugation in PPy films introduced by the COOH groups (Sheikhzadeh et al., 2016).

As the PAN/PPy/PPy3COOH NF mats prepared by immersing PAN NFs into the 20 wt% FeTos solution prior to VPP process were free of FeTos crystals and exhibited both a uniform coating and the lowest charge transfer resistance, they were chosen for further elaboration of the glucose biosensor.

### Analytical Performances of the Glucose Biosensor

In order to study the influence of Py/Py3COOH molar ratio in the monomers solution on the final glucose biosensor response, different electrodes were prepared using pure Py3COOH, and 1:1 or 2:1 Py3COOH/Py mixtures. GOx was covalently bound to the NFs after activation of the PPy3COOH carboxyl groups. Each electrode was measured by EIS in PBS solution in presence of different glucose concentrations in the 20 nM−2μM range.

Glucose could not be detected, neither with the PAN/ PPy3COOH/GOx NFs nor with the PAN/PPy/PPy3COOH/GOx NFs produced from 1:1 PPy/PPy3COOH solutions. The first result may be attributed to the too high density of carboxyl groups present on the surface, which may hamper activation reaction and/or subsequent binding of a sufficient amount of enzymes. The same issue and/or a slower evaporation rate of the Py3COOH monomer compared to PPy may explain the absence of signal observed for the second type of NFs. Pure PPy polymerizes at ambient temperature within 7 min, whereas for Py3COOH a longer polymerization time is required (15 min). By setting the ratio of Py/Py3COOH monomers at 1:2, EIS measurements enabled successful detection of glucose, thus validating the successful incorporation of carboxylic groups onto the NFs surface. The charge transfer resistance decreased upon the increasing concentration of glucose, confirming other published results (Ramanavicius et al., 2014). This may be attributed to the reaction of GOx enzyme with glucose, the latter being converted into gluconolactone, which is rapidly hydrolyzed into gluconate ions (Figure S2). No signal was detected from the PAN/PPy/PPy3COOH control electrode.

**Figure 7:**
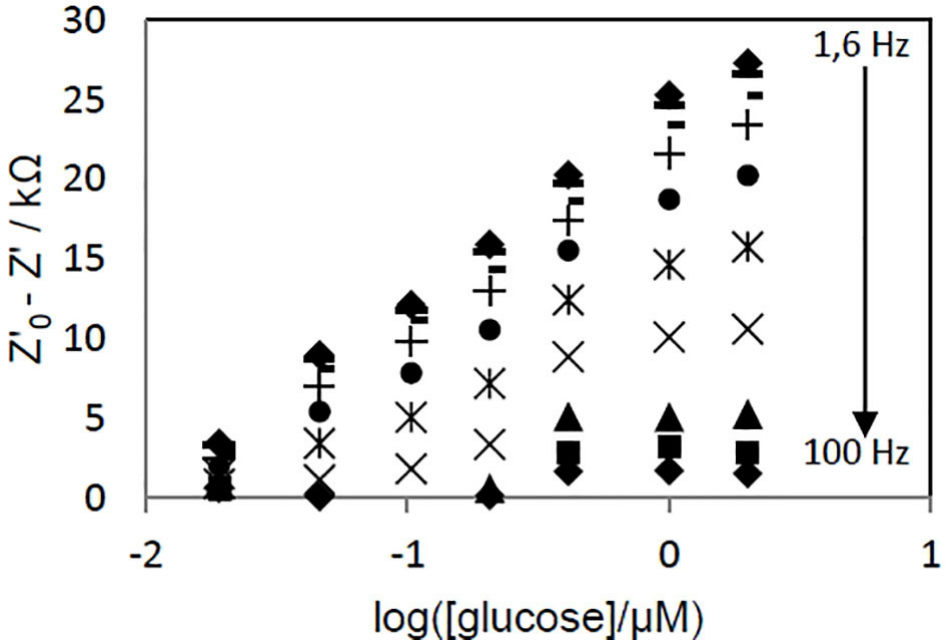
Effect of the applied voltage frequency on the evolution of PAN/FeTos 20wt%/PPy0.05M/PP3C0.1M/GOx NFs biosensor response with glucose concentration. EIS measurements performed at −300 mV vs Ag/AgCl in PBS solution (0.1M, pH 7.2).

**Figure 8:**
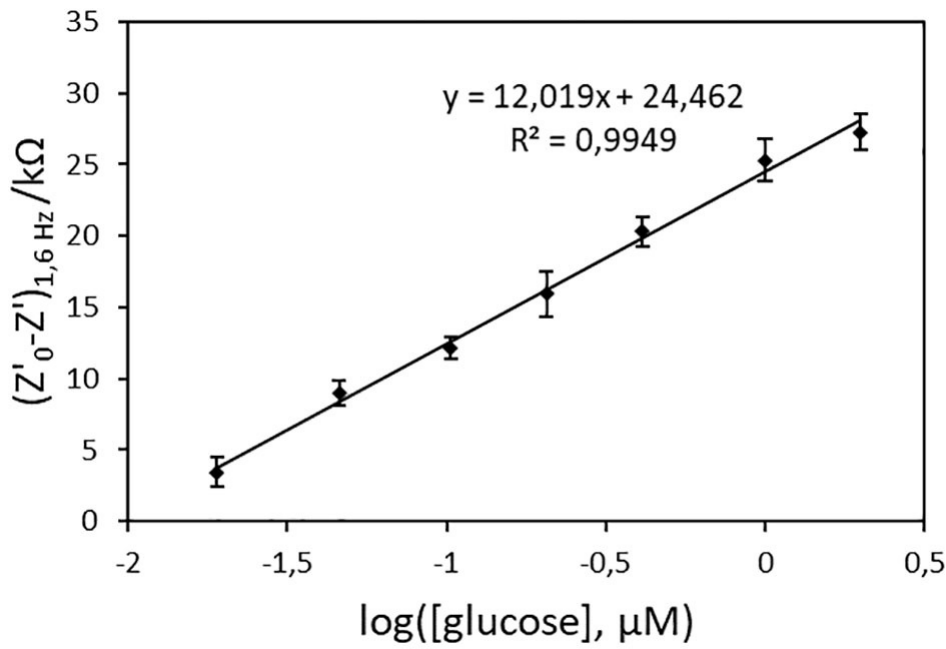
Calibration curve of the PAN/FeTos 20wt%/PPy/PPy3COOH/GOx NFs biosensor (EIS values at 1.6 Hz and −300 mV vs. Ag/AgCl in PBS 0.1M, pH 7.2). Error bars represent standard deviations obtained from three successive measurements.

To determine the optimal frequency to be used for glucose quantification, the real part of impedance (Z') was collected for each concentration point and different frequency values. Z'_0_-Z', where Z'_0_ refers to *Z*'-value for [glucose] = 0, was plotted vs. log[glucose]. Figure 7 shows the biosensor response curves obtained for frequencies in the 1.6–100 Hz range. The highest sensitivity, deduced from the slope of the linear part of the curves, was achieved at 1.6 Hz. At this frequency, the curve was linear in the whole range of concentrations (*r*^2^ = 0.9949, Z'_0_-Z'(kΩ) = 12.019 x log([glucose, μM]) + 24.462), Figure 8) and an excellent repeatability of the biosensor response, calculated from three consecutive measurements, was obtained. This value was therefore chosen as optimal frequency for further experiments.

In order to evaluate inter-sensor reproducibility, six electrodes coming from two batches of the VPP process were tested. The relative standard deviation, calculated from the analysis of a 50 nM glucose solution using the six different biosensors, was 15%. The limit of detection (LOD), defined as the concentration of glucose for which the relative standard deviation was 50%, varied only between 1.3 and 2.7 nM. Moreover, all sensors tested exhibited linearity within the same range (20 nM−2 μM), implying that even though the thickness of the conducting PPy/PPy3COOH coating during the VPP was not accurately controlled, the impact of this fact on the reproducibility demonstrated by the developed glucose biosensors was negligible.

As shown in Table 1, the proposed biosensor exhibited a much wider linear range and lower LOD than the two other reported glucose biosensors based on core-shell electrospun NFs produced by combining electrospinning and VPP processes (Ekabutr et al., 2013; Cetin and Camurluz, 2017). This LOD value enables for the determination of glucose in human serum (normal concentrations: 3.6 mM−6.1 mM range), after a large dilution of the sample, which is highly beneficial for the reduction of matrix effects.

**Table 1:** Comparison of the proposed biosensor with amperometric glucose biosensors based on GOx and NFs prepared by combining electrospinning and VPP process.

**Core NFs**	**Monomers for VPP**	**GOx fixation**	**Detection**	**LOD (μM)**	**Linear range (μM)**	**References**
PAN/MWCNTs/FeTos	Py	Adsorption	Amperometry			Ekabutr et al., 2013
			No mediator	15.5	250–6000	
			With mediator	980	125–7000	
PAN/FeCl_3_	Py	Cross-linking	Amperometry			Cetin and Camurluz, 2017
			+0.65 V	7.8	10–3500	
			−0.65 V	70	30–600	
PAN/FeTos	Py/Py3COOH (1:2)	Covalent binding	EIS	0.002	0.020-2	This work

Ascorbic acid and uric acids, which are classical interfering species in blood glucose determination, did not significantly modify the biosensor signal recorded for 100 nM glucose, when introduced at the 5 nM concentration. This glucose:interferent ratio was chosen as it is representative of the one found in blood serum samples.

No significant decrease of the PAN/PPy/PPy3COOH/GOx biosensor response was observed after 10 measurements of the 1 μM glucose solution, performed the same day. In the same way, EIS signal remained stable for 10 days when the biosensor was tested twice per day at the 1 μM glucose concentration and the biosensor was kept at 4°C between 2 days of measurement. The signal started to decrease slowly after 10 days, the signal drop being 15% after 1 month. The results demonstrate the good operational and storage stabilities of the PAN/PPy/PPy3COOH/GOx NFs biosensor.

Kausaite-Minkstimiene et al. proposed two amperometric glucose biosensors based on GOx covalently bound to PPyCOOH particles. In the first publication (Kausaite-Minkstimiene et al., 2018), the PPyCOOH-GOx composite was mixed with BSA and cross-linked at the surface of a graphite electrode. The influence of different parameters (pH of GOx solution for particles modifications, and pH of measurement electrolyte) on the biosensor signal was demonstrated. However, the addition of an electron-transfer mediator was required and the biosensor was not suitable for online glucose monitoring. A reagent-less amperometric biosensor based on GOx/PPyCOOH particles/gold nanoparticles composites was very recently developed by the same group (Kausaite-Minkstimiene et al., 2018), exhibiting a low limit of detection (0.08 mM), excellent stability and anti-interference ability and a wide linear range (0.2 M−150 mM). This biosensor is somewhat more easy to fabricate than the biosensor we propose. In addition, it has been shown that it is suitable for glucose determination in pharmaceutical preparations and human serum samples. However, the GOx/PPy/PPy3COOH/PAN NFs impedimetric biosensor, though perhaps not totally optimized, offers a lower limit of detection, which enables a larger dilution of samples.

## Conclusion

In the present study, we illustrated the successful elaboration of an original electrochemical glucose biosensor based on electrospun PAN NFs covered with conducting PPy/PPy3COOH coatings via VPP, followed by covalent grafting of GOx onto the surface of the NFs. Owing to the poor electrospinnability of PAN/FeTos solutions, immersion of PAN NFs into FeTos oxidant solution was found to be the best way to produce the PAN/FeTos oxidant NFs used as support for subsequent Py/Py3COOH polymerization.

The resulting glucose biosensor exhibited excellent analytical performances, by achieving linearity in a wide range of glucose concentration (20 nM−2 μM), good operational and storage stability, selectivity toward uric and ascorbic acids, and very low LOD values (2 nM) when compared to already reported biosensors. Biosensors coming from different batches exhibited linearity in the same concentration ranges thus implying good reproducibility of the VPP method. These results demonstrate the potential of the proposed approach for the development of new performing electrochemical biosensors. Glucose oxidase was chosen as model enzyme but carboxyl groups of the PPy/Py3COOH coating could be used to bind covalently other enzymes, or other sensing biomolecules such as aptamers, antibodies,…paving the way to a wider range of biosensing applications.

## Data Availability Statement

The raw data supporting the conclusions of this article will be made available by the authors, without undue reservation.

## Author Contributions

ES, J-FC, and FL designed experiments, interpreted data, and prepared the manuscript. ES did the experimental work. All authors contributed to the article and approved the submitted version.

## Conflict of Interest

The authors declare that the research was conducted in the absence of any commercial or financial relationships that could be construed as a potential conflict of interest.
