# Supplementary material for: Combining Electrospinning and Vapor-Phase Polymerization for the Production of Polyacrylonitrile/ Polypyrrole Core-Shell Nanofibers and Glucose Biosensor Application
Source: Front Chem. 2020 Aug 4;8:678. doi: 10.3389/fchem.2020.00678 (PMC7417620; doi:10.3389/fchem.2020.00678)
Supplement: Supplementary file 1 [file Data_Sheet_1.docx]

Supplementary Material

**(a)**

**(b)**

**(c)**

**Figure S1.** Evolution of the coloration of PAN NFs during the VPP procedure. a) Uncoated PAN fibers, b) FeTos coated PAN NFs after annealing at 70°C, c) PPy coated PAN NFs after 15min polymerization time at room temperature


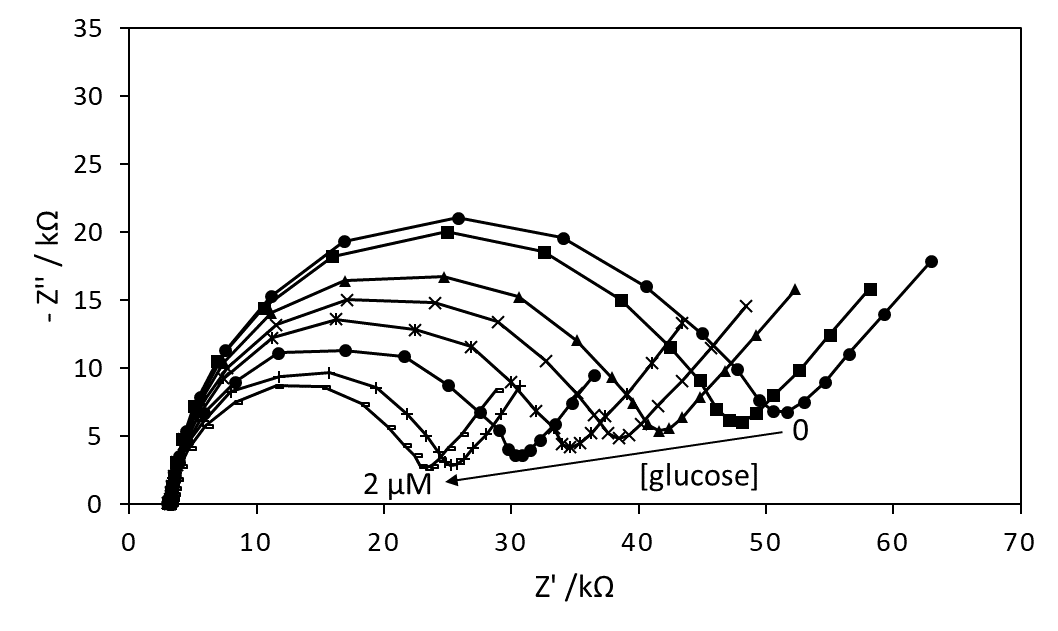


**Figure S2.** Nyquist plots of impedance spectra obtained for gold electrodes modified with PAN/FeTos 20wt%/PPy0.05M/PPy3COOH0.1M/GOx electrospun NFs upon the increasing concentration of glucose. EIS measurements performed at -300 mV in PBS solution (0.1M, pH 7.2), by varying frequency in the 100 mHz to 100 kHz range. Experimental points have been linked to improve the readability of the figure.
